# Tissue mechanics modulate PCNP expression in oral squamous cell carcinomas with different differentiation

**DOI:** 10.3389/fonc.2022.1072276

**Published:** 2023-01-10

**Authors:** Leyang Zhang, Dingcheng Guo, Junjie Shen, Yayuan Zheng, Junkai Zhai, Ruiping Li, Dengqi He, Baoping Zhang

**Affiliations:** ^1^ School (Hospital) of Stomatology Lanzhou University, Lanzhou, China; ^2^ The First Hospital of Lanzhou University, Lanzhou, China; ^3^ Gansu Province Key Lab of Maxillofacial Reconstruction and Intelligent Manufacturing, Lanzhou University, Lanzhou, China; ^4^ Gansu Provincial Clinical Research Center for Oral Disease, Lanzhou University, Lanzhou, China; ^5^ Institute of Biomechanics and Medical Engineering, Lanzhou University, Lanzhou, China; ^6^ Key Laboratory of Mechanics on Disaster and Environment in Western China, Ministry of Education, College of Civil Engineering and Mechanics, Lanzhou University, Lanzhou, China

**Keywords:** OSCC (oral squamous cell carcinoma), PEST-containing nuclear protein (PCNP), tissue interface mechanics, biomarker, prognosis

## Abstract

**Background:**

PEST-containing nuclear protein (PCNP), a novel zinc finger protein, participates in cell cycle regulation. Previous studies have confirmed that PCNP plays a role in mediating cellular development and invasion in a variety of cancer types. However, the relationship between PCNP expression and the occurrence and development of oral squamous cell carcinoma (OSCC) requires further exploration. In this study, we used biological atomic force microscopy to examine the histomorphological and mechanical properties of OSCC to explore the relationship between PCNP expression and differentiation of OSCC.

**Methods:**

Seventy-seven OSCC samples with varying degrees of differentiation were selected for hematoxylin and eosin staining, immunohistochemistry, and cellular mechanical measurement. The expression of PCNP and the mechanical properties such as stiffness and roughness of the tissue interface in OSCC samples were investigated. The Kaplan-Meier survival curve was utilized to assess the relationship of PCNP expression with patient survival.

**Results:**

The level of PCNP was significantly higher in well-differentiated OSCC than in moderately and poorly differentiated OSCC (P < 0.001). High expression of PCNP was specifically associated with higher tumor differentiation, lack of lymph node metastasis, and lower tumor node metastasis stage (all P < 0.05). Patients with high PCNP expression had a higher survival rate than those with low PCNP expression. The average variation of stiffness within a single tissue ranged from 347 kPa to 539 kPa. The mean surface roughness of highly, moderately, and poorly differentiated OSCC and paraneoplastic tissues were 795.53 ± 47.2 nm, 598.37 ± 45.76 nm, 410.16 ± 38.44 nm, and 1010.94 ± 119.07 nm, respectively. Pearson correlation coefficient demonstrated a positive correlation between PCNP expression and tissue stiffness of OSCC (R = 0.86, P < 0.001).

**Conclusion:**

The expression of PCNP was positively correlated with patient survival, tumor differentiation, and mechanical properties of tissue interfaces. PCNP is a potential biomarker for the early diagnosis and staging of OSCC. Furthermore, determination of the mechanical properties of the tissue interface could provide further useful information required for the detection and differentiation of OSCC.

## 1 Introduction

Oral and maxillofacial malignancies are among the most common malignancies of the head and neck. Oral squamous cell carcinoma (OSCC) accounts for approximately 90% of all oral malignancies (1), and its incidence is increasing (2). However, the prognosis of OSCC is poor (3). A survey conducted in 2020 reported that 177,757 of the 377,713 patients diagnosed with OSCC had died, and the 5-year survival rate was less than 50% (4). The treatment of OSCC is mainly based on the progression of the disease. Cancers in the early stage (stages I and II) can be treated with surgery or radiation. However, for patients with advanced OSCC (stages III and IV), it is difficult to achieve satisfactory therapeutic effects even when combining surgery, radiotherapy, and chemotherapy. Furthermore, early detection is difficult because of the location of OSCC. Therefore, patients are often diagnosed at an advanced stage of disease. Additionally, even after surgery and radiotherapy, more than half the patients develop local recurrence or distant metastasis, which lead to poor prognosis (5). Therefore, identifying biomarkers for the early diagnosis of OSCC is vital to identify necessary treatments and improve prognosis.

PEST-containing nuclear protein (PCNP) is a novel zinc finger protein that was first discovered in the nucleus through data mining (6). PCNP is involved in cell cycle regulation through its interaction with cell cycle regulatory proteins (7). Previous studies have demonstrated that the expression of PCNP in myeloma and central nervous system cancer was significantly higher than that of normal tissues, and overexpression of PCNP promotes the proliferation, migration, and invasion of lung adenocarcinoma cells (8). However, studies in neuroblastoma models have found that PCNP has tumor-suppressive effects (9). The level of PCNP expression in OSCC with varying degrees of differentiation remains unclear. Furthermore, the utility of PCNP in the diagnosis and choice of treatments of OSCC has not been investigated.

The biological processes of cancer cell proliferation, adhesion, differentiation, and apoptosis ultimately depend on mechanical behaviors, such as mechanical force transmission and motion deformation by microfibers of the cytoskeleton (10). Therefore, cell mechanics can be used to investigate the characteristics of cancer cells at microscale and nanoscale levels. Biomechanical studies showed that changes in cell mechanical properties, such as stiffness, cell elasticity, viscoelasticity, and membrane surface adhesion energy, could be a novel method of characterizing cancerous cells (11). Abnormal cell stiffness is closely related to the occurrence and development of cancer (12, 13). Invasive cancer cells are relatively soft, which reduces cell adhesion to the extracellular matrix and enhances the ability of cells to detach from carcinoma in situ. This process is the result of interactions between tumor cells, stromal cells, and the extracellular matrix (14). Biological atomic force microscopy (Bio-AFM) is a powerful tool, particularly at microscale- and nanoscale levels (15). It is widely used in biomedical research to determine the mechanical properties of various tumor tissues including in breast cancer (16), liver cancer (17), lung cancer (18), bladder cancer (19), colon cancer (20), head and neck cancer (21), and prostate cancer (22). It is used to explore the intrinsic relationship between tumor cell proliferation, adhesion, metastasis, and the mechanical properties of tumor cells. In this study, we used Bio-AFM to examine the histomorphological and mechanical properties of OSCC, to explore the relationship between PCNP expression and differentiation of OSCC. We hope to develop strategies for the early and accurate diagnosis and prognostic prediction of OSCC.

## 2 Methods

### 2.1 Tissue preparation

The research protocol was approved by the Research Ethics Committee of the First Hospital of Lanzhou University (No. LDYYLL-2022-321). After obtaining written informed consent, we collected tumor samples from 77 patients with OSCC registered in the Department of Oral and Maxillofacial Surgery, between February 2017 and October 2021. These included well, moderately, and poorly differentiated tumors. Samples from 50 men (65%) and 27 women (35%), aged 37-86 years (mean age 62.1 years) were included in the study. Forty-eight patients did not have lymph node metastasis and 29 had cervical lymph node metastasis.

Following the maxillofacial surgery, cancerous and cervical lymph node tissues were harvested. All specimens were taken from typical lesions and fixed in 10% neutral-buffered formalin before being paraffin-embedded. Tumor biopsy was followed by pathological analysis by two experienced pathologists to rule out other diseases (including inflammation at other sites and secondary tumors). Clinical tumor node metastasis (TNM) staging was performed according to the 7th edition of the TNM staging classification criteria jointly developed by the International Union for Cancer Control and American Joint Committee on Cancer (23) and the World Health Organization guidelines (24).

### 2.2 Hematoxylin and eosin staining

Tissues were fixed in 4% neutral paraformaldehyde overnight, embedded in paraffin, and cut into 4-μm-thick slices, which were deparaffinized with xylenes and rehydrated in an ethanol gradient. The slices were stained with hematoxylin for 5 minutes, then washed with hydrochloric acid and ethanol, and stained in eosin for 3 minutes. Following this, the slides were subjected to gradient dehydration, transparency, sealing, and neutral resin sealing. An Olympus BX53 microscope was used to visualize and image the slices at 10×, 20×, and 40× magnifications.

### 2.3 Immunohistochemistry

The sections were cut, dried, dewaxed and hydrated. Then incubated it with rabbit anti-human polyclonal PCNP antibody (1:200, batch No: 11180-2-ap) at 4°C overnight and followed by a secondary antibody (HistostainTM-Plus Kits, SP-9001) at 37°C for 1h. DBA was used for the dehydration, transparent, film, and neutral resin sealing steps. Then visualized staining under the Olympus BX53 microscope. Samples were given scores above 4 and considered as “high” expression (25).

### 2.4 Reverse transcription-polymerase chain reaction

For RNA extraction, the MolPure^®^ Cell/Tissue Total RNA Kit (19221ES50) was utilized, and Hifair^®^ III SuperMix (11141ES60, 100T) was employed for reverse transcription of the first-strand complementary DNA. QuantiNova SYBR Green PCR Kit (208054) was used for reverse transcription-polymerase chain reaction (RT-qPCR). The following primers were used to detect PCNP expression: PCNP-F: CCAGTGGTCTTGGTGTGCTG and PCNP-R: AGCTCCGTGAAGACCTGGAG. The internal reference gene was GAPDH. All results are relative expression levels determined using the 2^-ΔΔCT^ method.

### 2.5 Bio-AFM tissue mechanics

The mechanical properties of the tissues were determined using a biological atomic force microscope (Nano Wizard III, Bruker). All specimens were taken from typical lesions and gently fixed in 4% neutral paraformaldehyde for 10 min. Then the sections were cut into 10-μm thick slices and placed on a mica. All the process were finished by the same experimenter. The AFM probes were Multi75DLC (Diamond-Like-Carbon coating Budget Sensors, Bulgaria) with a 3 N/m force constant. AFM probe specifications are shown in **Table S1**. Before measuring, the probe’s spring constant was calibrated using the thermal vibration that was integrated into the device. AFM was then performed using a contact model with a 0.5 Hz/s scanning rate. When measuring the structure, morphology, and mechanical characteristics of the sample while probing, the force-distance curve was constructed with a velocity of 5 μm/s in contact with the tissue. For each sample we selected 15 random sites, and each site was measured 10 times. The improved Hertz/Sneddon model was utilized to assess the force-distance curve for each slide (26) and calculated the Young’s modulus and roughness of OSCC tissues with different degrees of differentiation using JPK data processing software (version 7.0.97).

### 2.6 Statistical analysis

Statistical analysis was performed using SPSS 26.0 (IBM, America). The data are represented as mean with standard error and statistically analyzed using one-way ANOVA or, for paired comparisons, the Tukey-Kramer HSD test. The correlation between clinical features and PCNP expression were examined by Pearson’s chi-square test and Fisher’s exact test based on the computed odds ratios and 95% confidence intervals. Kaplan-Meier curves were used to measure survival, and log-rank tests were used to investigate differences. The significance level was chosen at P < 0.05.

## 3 Results

### 3.1 OSCC clinical stage, histomorphology, and PCNP expression

**Figure 1:**
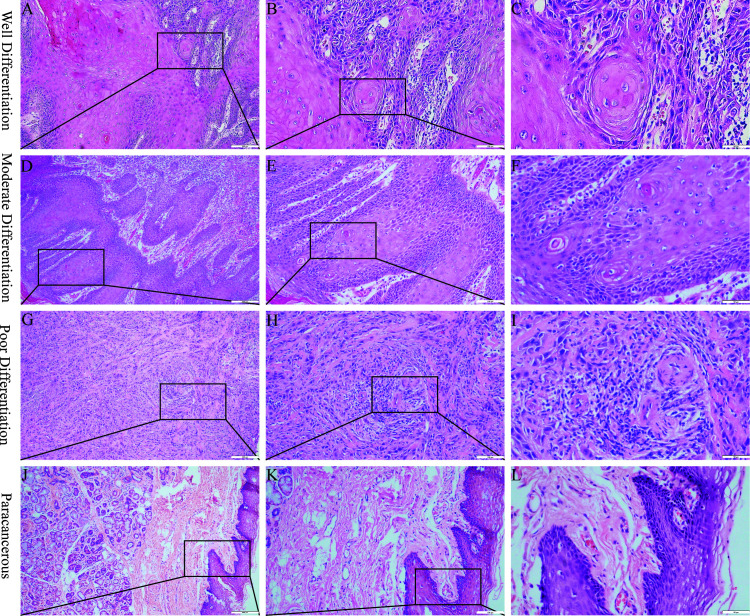
Hematoxylin and eosin staining of OSCC. **(A–C)** Well differentiation OSCC in 10×, 20×, 40×. **(D–F)**, Moderate differentiation OSCC in 10×, 20×, 40×. **(G–I)** Poor differentiation OSCC in 10×, 20×, 40×. **(J–L)** Paracancerous samples in 10×, 20×, 40×.

Representative samples of poorly, moderately, and well-differentiated OSCCs are shown in **Figure 1**. Poorly differentiated OSCC consisted mainly of immature cells, with a large number of normal or abnormal nuclear divisions. There was very little keratosis and few intercellular bridges. Similar to poorly differentiated OSCC, moderately differentiated OSCC was characterized by polymorphonuclear and nuclear division. Keratosis was uncommon in moderately differentiated OSCC, and there were few intercellular bridges. Well-differentiated OSCC was similar to normal squamous epithelium and contained basal and squamous cells with intercellular bridges. It had obvious keratosis, rare abnormal nuclear division, and an inconspicuous polytypic nature of the nucleus and cell.

**Figure 2:**
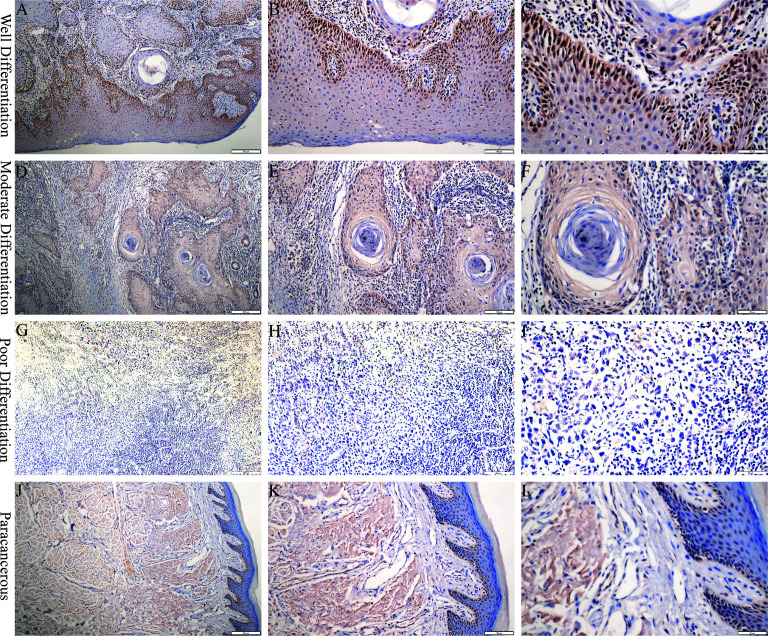
Immunohistochemical staining was performed to detect the expression of PCNP at different stages of OSCC. **(A–C)** Well differentiation OSCC in 10×, 20×, 40×. **(D–F)** Moderate differentiation OSCC in 10×, 20×, 40×. **(G–I)** Poor differentiation OSCC in 10×, 20×, 40×. **(J–L)** Paracancerous samples in 10×, 20×, 40×.

**Figure 3:**
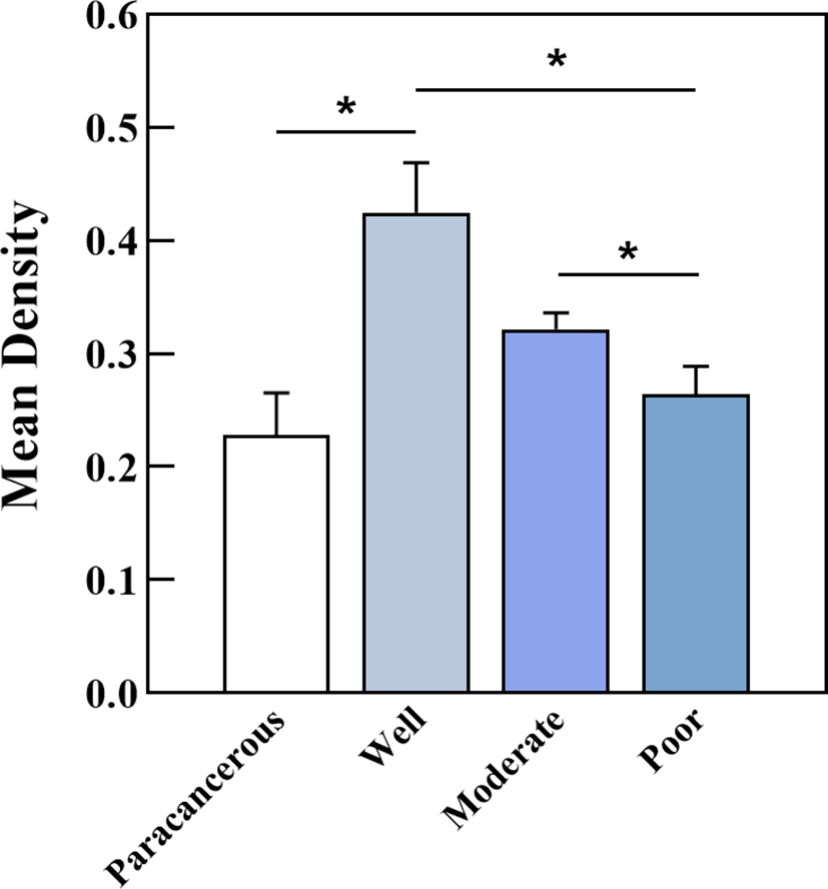
Average optical density of PCNP in well, moderately, and poorly differentiated OSCC. Well-differentiated OSCC had high expression in IHC (*P < 0.05).

**Figure 4:**
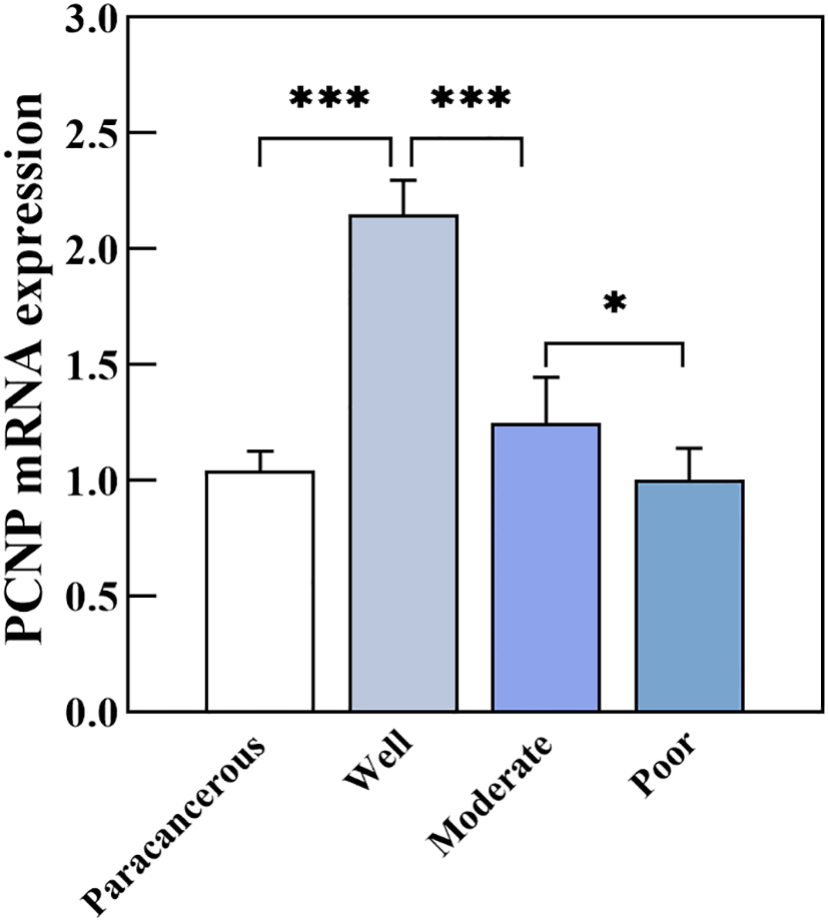
PCNP mRNA expression in well, moderately, and poorly differentiated OSCC (*P < 0.05, ***P < 0.001).

PCNP was primarily located in the nucleus of tumor cells but not in neighboring normal epithelial cells. PCNP was highly expressed in well-differentiated OSCC and expressed at low levels in poorly differentiated OSCC, but both had higher levels than paracancerous tissues (**Figure 2**). The average optical density values of PCNP were 0.424 ± 0.044, 0.322 ± 0.014, 0.264 ± 0.024, and 0.2228 ± 0.037 in well, moderately, and poorly differentiated OSCC and paracancerous tissues, respectively (P < 0.001) (**Figure 3**). These results were supported by RT-qPCR, which showed that well-differentiated OSCC tissues had higher levels of PCNP expression than moderately and poorly differentiated OSCC tissues, and cancer tissues had higher levels of PCNP expression than paracancerous tissues (**Figure 4**).

Further, we investigated the clinicopathological features of OSCC in varying stages (**Table 1**). PCNP expression was closely correlated with the degree of tumor differentiation, TNM clinical stage, as well as lymph node metastasis (P < 0.05). Additionally, logistic regression analysis revealed that high PCNP expression was a protective factor against lymph node metastasis, distant metastasis, and TNM staging. In comparison to high differentiation, PCNP expression was a protective factor against moderate and poor differentiation. Compared to patients with poorly and moderately differentiated tumors, those with well-differentiated tumors had considerably higher PCNP levels (P < 0.05), and patients with lymph node metastases had lower PCNP levels than patients without lymph node metastases. Additionally, patients with more advanced TNM stages had lower levels of PCNP than those with earlier TNM stages. However, there was no association between PCNP expression level and tumor size or sex (P > 0.05).

**Table 1:** Relationship between PCNP expression and clinical features of OSCC.

Characteristic	n	PCNP expression		Pearsonχ^2^	>P value	>ORs
		Low or no	Positive			
Total	77	22	55			
Sex				0.143	0.706	
Male	50	15	35			
Female	27	7	20			
Age				0.520	0.471	
≥60 years	40	10	30			
<60 years	37	12	25			
Localization				0.896	0.826	
Lip	18	4	14			
Tongue	26	9	17			
Gingiva	12	3	9			
Other	21	6	15			
Differentiation				14.831	0.001	
Well	36	3	33			
Moderate	25	10	15			0.136
Poor	16	9	7			0.071
Tumor stage					0.548	
T1\T2	60	16	44			
T3\T4	17	6	11			
Lymph node Metastasis				6.024	0.014	0.284
Positive	29	13	16			
Negative	48	9	39			
Distant metastases					0.286	0.276
M0	76	21	55			
M1	1	1	0			
TNM stage				4.107	0.043	0.353
I+II	42	8	34			
III+IV	35	14	21			

### 3.2 PCNP expression and patient survival

**Figure 5:**
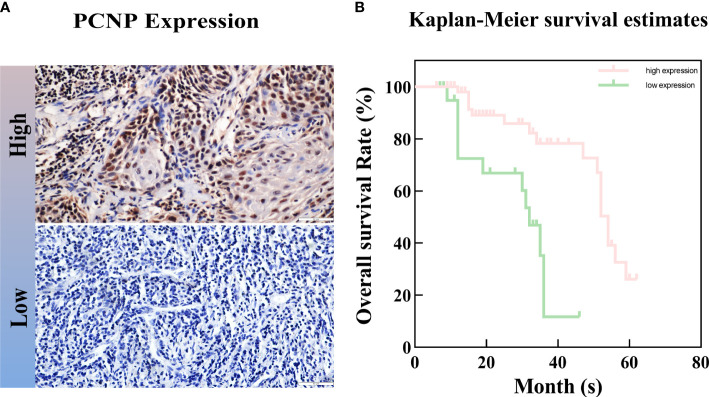
Kaplan-Meier survival curves based on PCNP expression (low expression, green line; high expression, pink line). **(A)** High and low expression of PCNP. **(B)** Kaplan-Meier survival curves.

Using Kaplan-Meier survival curves, we found that PCNP expression was likely to be related to overall survival in patients with OSCC (**Figure 5**). The PCNP-low expression group had a 72.4% 1-year survival rate, whereas the PCNP-high expression group had a 98.0% 1-year survival rate. The 3-year survival rates were 11.7% in the low expression group and 72.6% in the high expression group.

### 3.3 OSCC tissue surface morphology and roughness

**Figure 6:**
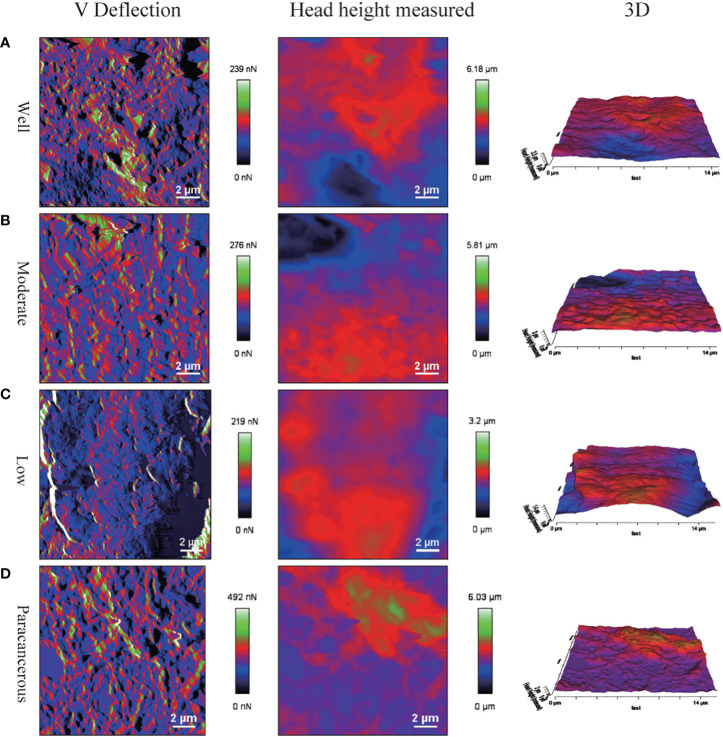
Surface morphology of OSCC tissue sections via AFM detection. Poorly differentiated tissues had irregular morphology. **(A)** V Deflection, Head height measured and 3D images of well differentiation OSCC. **(B)** V Deflection, Head height measured and 3D images of moderate differentiation OSCC. **(C)** V Deflection, Head height measured and 3D images of poor differentiation OSCC. **(D)** V Deflection, Head height measured and 3D images of paracancerous samples.

**Figure 7:**
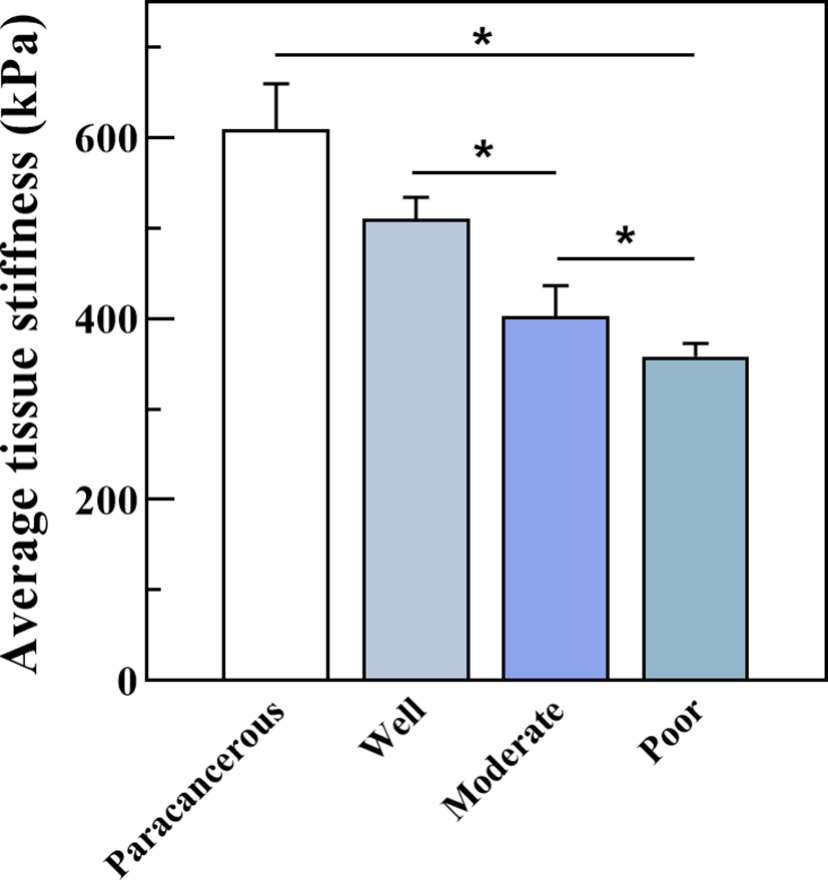
Surface roughness of well, moderately, and poorly differentiated OSCC. Results represent the mean ± SD in histogram (*P < 0.05).

The surface morphology of OSCC tissues with various degrees of differentiation was directly imaged and analyzed using Bio-AFM. A representative image of a sample of each differentiation obtained during the cantilevered AFM nanoindentation test is shown in **Figure 6**. The tissue interface varied based on the tumor differentiation. Well-differentiated OSCC tissues were regular and flat, whereas poorly differentiated OSCC tissues had an overall irregular morphology with obvious modulation and loose tissue. The roughness of the tissue surface was enhanced with better OSCC tissue differentiation (**Figure 7**). The mean surface roughness of well, moderately, and poorly differentiated OSCC and paraneoplastic tissues were 795.53 ± 47.2 nm, 598.37 ± 45.76 nm, 410.16 ± 38.44 nm, and 1010.94 ± 119.07 nm, respectively.

### 3.4 Mechanical properties of the OSCC tissue interface

**Figure 8:**
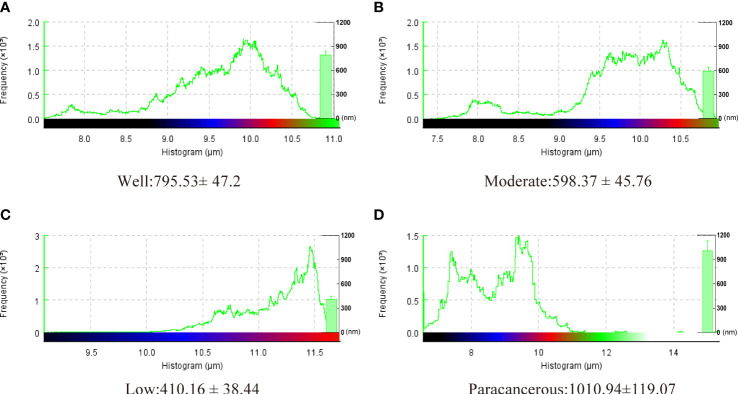
Average tissue stiffness tested by Bio-AFM. Well-differentiated OSCC had a higher Young’s modulus than moderately and poorly differentiated OSCC (kPa, P < 0.05).

**Figure 9:**
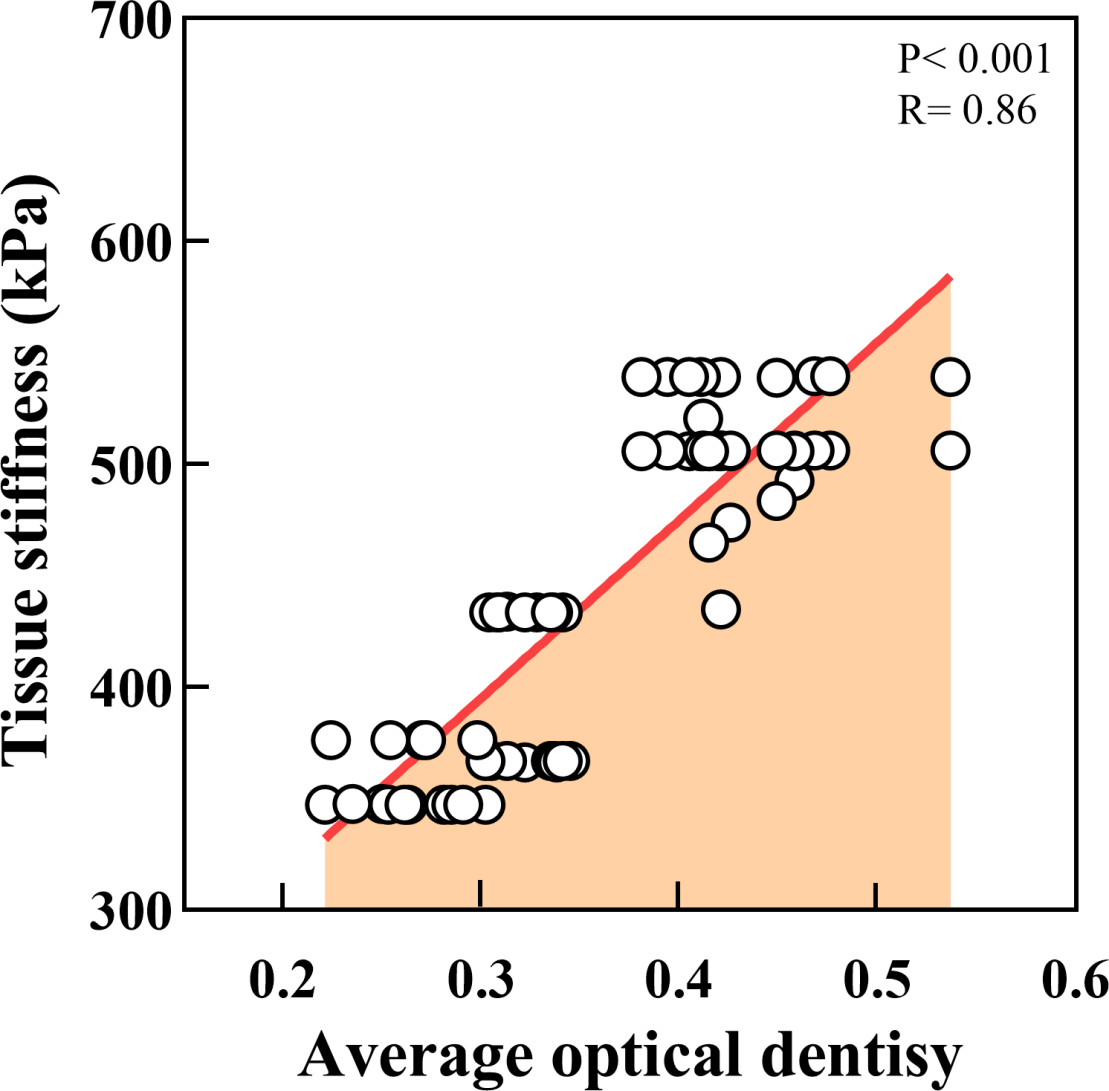
Correlation analysis between tissue stiffness of OSCC and PCNP expression. (P < 0.001, R = 0.86).

We further measured the Young’s modulus of the OSCC tissues. The average variation of stiffness within a single tissue ranged from 347 κPa to 539 κPa, in which well was 510.61κPa, moderate was 404.10κPa, and poor was 358.14κPa (**Figure 8**). Poorly differentiated samples had lower stiffness than well- and moderately differentiated samples (P < 0.01). The representative plots of force-distance curves for each group of samples are shown in **Figure S1**, and the distribution of stiffness in all groups of samples is demonstrated in **Figure S2**. The Pearson correlation coefficient also determined a positive correlation between PCNP protein level and OSCC tissue stiffness (R = 0.86, P < 0.001) (**Figure 9**), which demonstrated that as the degree of tumor differentiation increased, tissue stiffness increased accordingly.

## 4 Discussion

PCNP is involved in cell cycle regulation, and high levels of PCNP have been found in various cancer cell lines, including U-937 myeloid leukemia cells and HepG2 hepatocellular carcinoma cells, suggesting that PCNP may be engaged in carcinogenesis (27). However, PCNP’s role and mode of action in OSCC remains unknown. We discovered that PCNP is a potential marker of the presence of OSCC cells and tissues. Our study revealed that PCNP expression was significantly higher in well-differentiated OSCC tissues than in moderately and poorly differentiated tissues, and cancer tissues expressed more of the protein than paracancerous tissues. In addition, PCNP levels were higher in patients with an early TNM stage than in those with an advanced TNM stage. These findings may indicate that PCNP expression may have utility in the diagnosis, staging and prognosis of OSCC.

A previous study reported that PCNP had an inhibitory role in neuroblastoma cell processes such as growth, migration and invasion through increased ratios of Bad/Bcl-xl and Bax/Bcl2 and upregulation of Caspase-3, 8, and 9 (11), in which the PI3K/AKT/mTOR signaling cascade also played a crucial role. This signaling pathway is not only essential for cell growth, survival, motility, and protein transport but also contributes to the onset and progression of OSCC. PI3K inhibitors can effectively suppress the development of OSCC cells by inhibiting PI3K/AKT/mTOR signaling (28). In addition, overexpression of PCNP decreased human thyroid cancer cell proliferation, migration, invasion and xenografts, affected apoptosis by activating the ERK/JNK/p38 pathway, and influenced cell cycle arrest by altering the expression of genes that regulate the cell cycle (29). Instead, another study showed that high level PCNP regulates angiogenesis and facilitates migration, proliferation, and invasion and adenocarcinoma xenografts in lung cancer (9) and colon cancer (30). Furthermore, Dong et al. (31) demonstrated that PCNP enhances ovarian cancer by interacting with β-catenin and promoting its nuclear translocation. Therefore, it appears that PCNP affects various tumor types differently, which may be related to tumor heterogeneity. Additionally, the expression of tumor necrosis induced protein 8-like 2 (TIPE2) and PCNP in peripheral blood mononuclear cells of active rheumatoid arthritis (RA) patient has abnormally increased, and there was a positive correlation between them (30). TIPE2 was determined to serve as a negative regulator of macrophages and T cells *via* regulating the expression and function of toll-like receptor (TLR) and T cell receptor (TCR) (32) and the inhibitor of the mitogen-activated protein kinase (MAPK) and nuclear factor κ-light chain-enhancer of activated B cells (NF-κB) signaling pathways (33, 34), in the same time inhibit the immune response at the time of inflammation in recent researches (35–37). These may imply the correlation between PCNP and immune system and diseases.

Studies on OSCC have mainly focused on its molecular, microbiological, immunological, and pathological aspects, but there are few reports on its biomechanical aspects (38). The application of biomechanical methods may further improve the accuracy of early diagnosis of OSCC. As a novel technique in tumor research, AFM is currently garnering interest. When tumors grow, the cytoskeleton undergoes modifications, and these alterations in the cytoskeleton have a knock-on effect on the adhesion, stiffness, and roughness of the cells (39). A number of pertinent studies have demonstrated that Young’s modulus can reflect the hardness of malignant cells or tissues, which are softer than normal tissue or cells. As a result, Young’s modulus is also thought to be an indication of changed cancerous tissue (40). The Young’s modulus of the tumor tissue in clear cell renal cell carcinomas was much lower than that of normal tissue, and the tumor tissue was significantly less fibrotic (41). Cancer cell adhesion, stiffness, and other mechanical characteristics also change as the disease develops (42). Laika et al. (43) measured the Young’s modulus of normal cells and human bladder cancer cells using AFM and found that the stiffness of cancer cells was one-tenth that of normal cells. By measuring the interfacial mechanical characteristics of OSCC tissues, we found that, compared with well-differentiated OSCC tissues, poorly differentiated OSCC tissues were looser in structure, softer than paracancerous tissue, and the range of the average stiffness variation within a single tissue was 347 kPa to 539 kPa. Those with poor differentiation demonstrated less stiffness than samples with good and moderate differentiation. Their surface roughness was lower, poorly differentiated OSCC had a mean surface roughness of 410.16 ± 38.44 nm, whereas well-differentiated OSCC had a mean surface roughness of 795.53 ± 47.2 nm. This may be related to the different cytoskeletal arrangement morphology of tumor cells with different degrees of differentiation. We also assessed the tissue’s surface roughness, which followed the same trend as the stiffness of the tissue. This implies it may be a useful indicator cancer progression. However, the specific mechanism needs further investigation. Notably, the PI3K pathway is crucial to OSCC’s growth, and several medications decrease the multiplication of cancer cells by blocking the PI3K/AKT/mTOR signaling pathway (44–46). In addition, the PI3K pathway can affect the invasion and migration of tumor cells by reshaping the actin cytoskeleton. The small GTPase subfamily proteins RAC 1, 2, and 3 in the Rho family play a very important role in this process (47, 48). However, compared to normal tissue, Young’s modulus is frequently higher in cancer tissues (42, 49). The mechanical characteristics of human brain tissues (medulloblastoma, grade IV) were examined by AFM, and the results showed that these tissues are significantly heterogeneous, with values spread between 1.89 and 75.69 kPa and a mean of 27.16 kPa, which is higher than normal brain tissues (50). These conflicting findings may be a result of the heterogeneity of various tumor tissues.

Currently, pathological diagnosis is still the “gold standard” for tumor diagnosis (51); however, since the establishment of biomechanics two decades ago (52), biomechanical methods have been used to study the apoptosis, migration, proliferation, and differentiation of tumor cells (53), which has allowed for the development of new therapeutic methods. Increasing attention has been paid to the study of cancer by combining biomedical and biomechanical methods (54). Therefore, we aimed to provide a new perspective for the early diagnosis of OSCC by combining traditional diagnostic methods with biomechanical assessments. In this study, we utilized AFM to examine changes in the mechanical properties of OSCC tissues at the micro- and nanoscales, as well as the relationship between the degree of differentiation and tumor stiffness.

In summary, our research reveals a correlation between PCNP expression and biomechanical and clinical characteristics of OSCC. High PCNP levels were associated with higher overall patient survival. Additionally, high PCNP expression was associated with increased tumor stiffness and roughness and decreased migration and invasion. This study has some limitations which must be noted. First, this study was of a small sample size. Second, we did not examine the precise mechanism by which PCNP affects OSCC, which requires examination in future studies. A larger patient cohort, as well as more molecular and cellular biology studies, will aid in the validation of our findings and the establishment of PCNP as a precise and useful biomarker for the diagnosis and prognosis prediction of OSCC. We believe that using biomechanical methods can provide a **Supplementary Method** in the accurate diagnosis of OSCC.

## Data availability statement

The original contributions presented in the study are included in the article/**Supplementary Material**. Further inquiries can be directed to the corresponding authors.

## Ethics statement

The studies involving human participants were reviewed and approved by Research Ethics Committee of First Hospital of Lanzhou University. The patients/participants provided their written informed consent to participate in this study. Written informed consent was obtained from the individual(s) for the publication of any potentially identifiable images or data included in this article.

## Author contributions

LZ, DG, and JS: software, validation, formal analysis, investigation, writing-review and editing. YZ: data curation, writing-original draft. JZ: writing-review and editing, visualization. RL: supervision, project administration. DH and BZ: reviewed the manuscript and provided the conceptualization, methodology and funding acquisition. BZ handled correspondence at all stages of review and publication and will continue to handle it after publication. All authors contributed to the article and approved the submitted version.
