# Supplementary figures and images for: Tissue mechanics modulate PCNP expression in oral squamous cell carcinomas with different differentiation
Source: Front Oncol. 2023 Jan 10;12:1072276. doi: 10.3389/fonc.2022.1072276 (PMC9873348; doi:10.3389/fonc.2022.1072276)

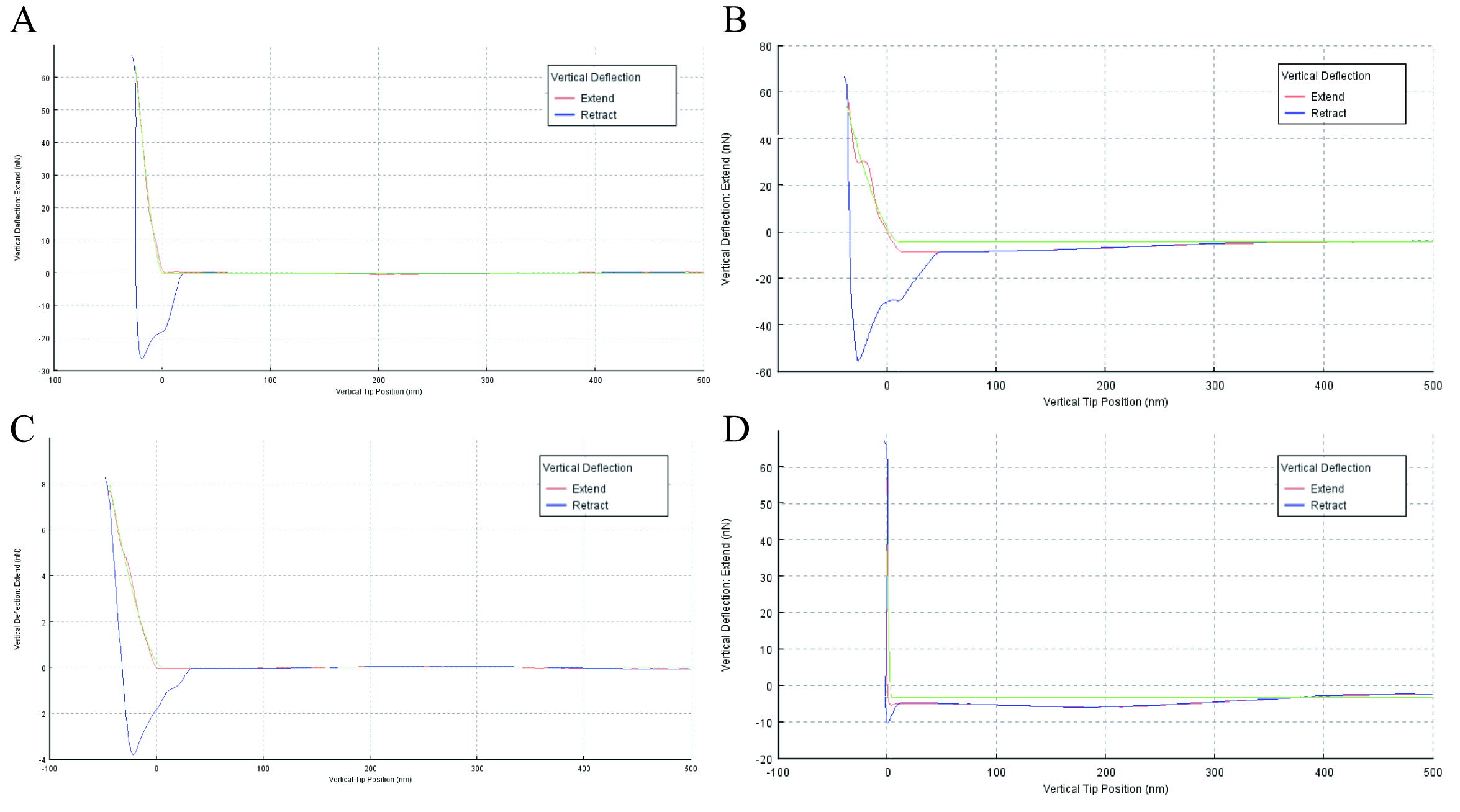

Supplement: Supplementary file 1 [file Image_1.tif]

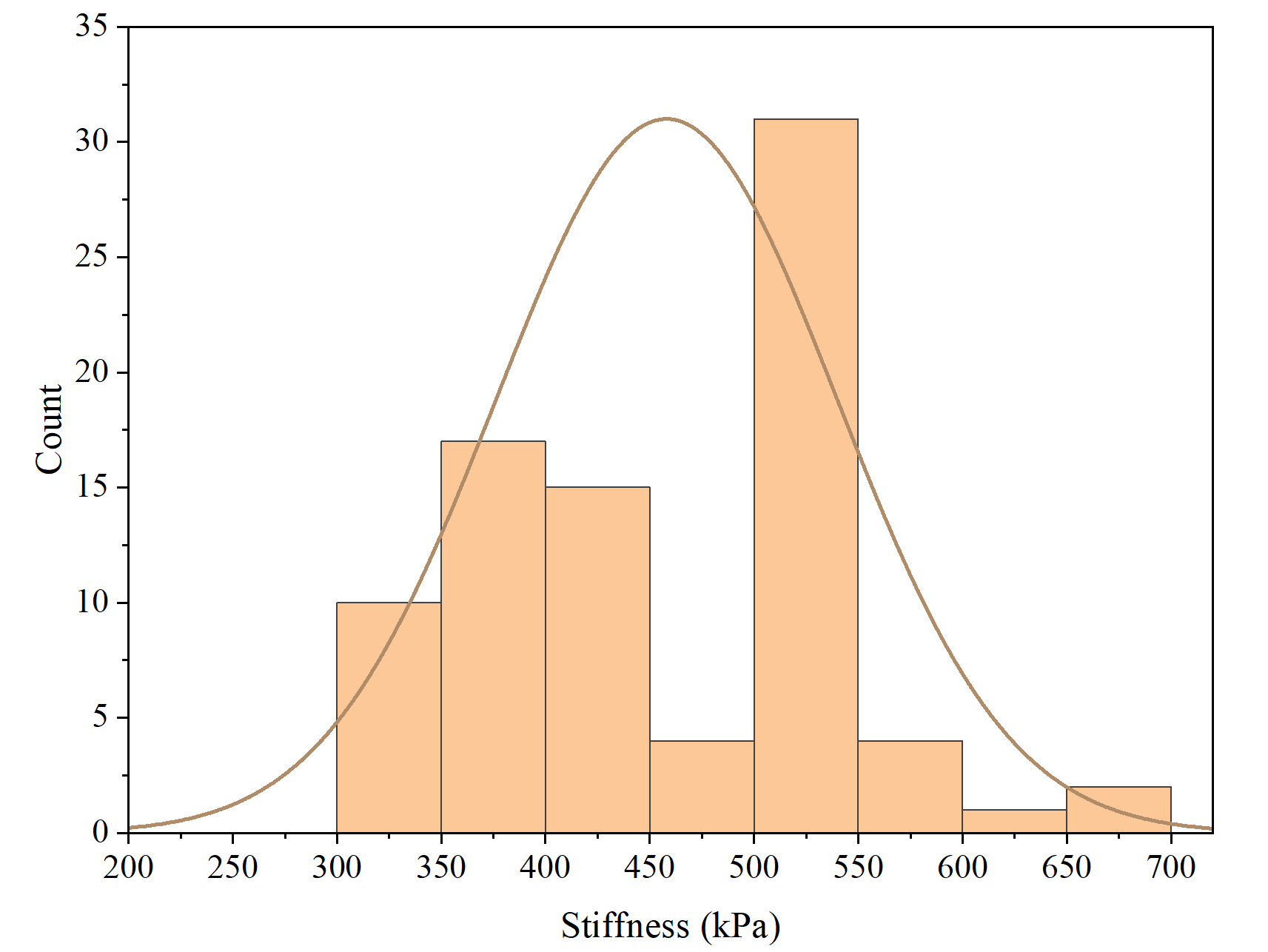

Supplement: Supplementary file 2 [file Image_2.tif]
