# Supplementary material for: Tissue mechanics modulate PCNP expression in oral squamous cell carcinomas with different differentiation
Source: Front Oncol. 2023 Jan 10;12:1072276. doi: 10.3389/fonc.2022.1072276 (PMC9873348; doi:10.3389/fonc.2022.1072276)
Supplement: Supplementary file 3 [file Table_1.docx]

Table S1. Geometrical properties of AFM tip and Cantilever.

**AFM Tip**

| Shape | Height | Setback | Radius | Half Cone Angle |
| --- | --- | --- | --- | --- |
| Rotated | 17µm | 15µm | 15nm | 25°-30° from side, 10° at the apex, 20°-25° along cantilever axis |

### AFM Cantilever

| Shape | Force Constant | Resonance Frequency | Length | Width | Thickness |
| --- | --- | --- | --- | --- | --- |
| Beam | 3N/m | 75kHz | 225µm | 28µm | 3µm |
